# Wafer-scale 3D shaping of high aspect ratio structures by multistep plasma etching and corner lithography

**DOI:** 10.1038/s41378-020-0134-6

**Published:** 2020-03-23

**Authors:** Shu Ni, Erwin J. W. Berenschot, Pieter J. Westerik, Meint J. de Boer, René Wolf, Hai Le-The, Han J. G. E. Gardeniers, Niels R. Tas

**Affiliations:** 1 Mesoscale Chemical System Group, MESA+ Institute, University of Twente, 7522 NB Enschede, The Netherlands; 2 Inorganic Materials Science Group, MESA+ Institute, University of Twente, 7522 NB Enschede, The Netherlands; 3 NanoLab Cleanroom, MESA+ Institute, University of Twente, 7522 NB Enschede, The Netherlands; 4 Physics of Fluids Group, MESA+ Institute & Max Planck Center for Complex Fluid Dynamics, University of Twente, 7522 NB Enschede, The Netherlands; 5 BIOS Lab-on-a-Chip Group, MESA+ Institute & Max Planck Center for Complex Fluid Dynamics, University of Twente, 7522 NB Enschede, The Netherlands

**Keywords:** Engineering, Nanoscience and technology

## Abstract

The current progress of system miniaturization relies extensively on the development of 3D machining techniques to increase the areal structure density. In this work, a wafer-scale out-of-plane 3D silicon (Si) shaping technology is reported, which combines a multistep plasma etching process with corner lithography. The multistep plasma etching procedure results in high aspect ratio structures with stacked semicircles etched deep into the sidewall and thereby introduces corners with a proper geometry for the subsequent corner lithography. Due to the geometrical contrast between the gaps and sidewall, residues are left only inside the gaps and form an inversion mask inside the semicircles. Using this mask, octahedra and donuts can be etched in a repeated manner into Si over the full wafer area, which demonstrates the potential of this technology for constructing high-density 3D structures with good dimensional control in the bulk of Si wafers.

## System miniaturization: Shaping with multistep plasma etching and corner lithography

A novel 3-D silicon shaping technology has been developed that combines a multistep etching process with corner lithography. Currently, system miniaturization relies extensively on 3-D machining techniques. A team headed by Shu Ni and Niels R. Tas at the University of Twente, Netherlands developed a versatile fabrication technique for wafer-scale micro- and nano-machining using a combination of multistep etching and corner lithography (layer deposition and subsequent timed etching of the layer, resulting in a well-defined residue in concave corners and layer removal on flat surfaces and convex corners). The multistep etching creates high-aspect-ratio structures with stacked semicircles etched deeply into the sidewall; corners are introduced with proper geometry for the subsequent corner lithography. The authors believe their technique has excellent potential for constructing high-density 3-D structures with good dimensional control in most silicon wafers.

## Introduction

Top-down bulk silicon (Si) fabrication is mostly performed by photolithography and subsequent etching to transfer patterns into Si substrates, forming etched two-dimensional (2D) features. These techniques were originally developed for integrated circuit (IC) and microelectromechanical (MEMS) devices, and later, they have been applied to microfluidics, energy conversion, and storage technologies^1^. Although top-down fabrication can provide precise control over features in-plane (*x* and *y* direction) using photolithography, it usually results in a projection of 2D shapes into the bulk of the substrates by using (an)isotropic or directional etching methods. Over the years, numerous three-dimensional (3D) technologies, such as inkjet printing^2^ and stereo-lithography^3^ have emerged, making use of third dimension construction to significantly improve the density of patterned features. Because these techniques rely on voxel-by-voxel processing in a serial writing mode, they are inefficient for wafer-scale production. Therefore, it is of great importance to develop versatile 3D fabrication techniques that are able to form 3D architectures using parallel wafer-scale micro- and nanomachining. Cerofolini et al.^4,5^ reported the realization of repeated structures on top of a Si wafer. However, this technique requires the deposition of as many alternating layers as the number of repetitions required. Moreover, the etching of vertical holes through these layer stacks is challenging because of the inconsistent etching behavior of different materials. In addition, due to the deposition rate of the layers, the height of the resulting structure is limited to a few micrometers. Furthermore, Joseph et al.^6^ and Luo et al.^7^ reported techniques for precise 3D shaping of grown Si nanowires by modifying the etching selectivity of specific locations across these nanowires. This can be done either by locally controlling the concentration of phosphorous (P)—an n-type substitutional dopant with high solubility in Si—in the Si nanowires^6^ or by manipulating the gold diffusion along the Si nanowire sidewalls, as metal can diffuse along semiconductor surfaces and change the surface behavior^7^.

By extending one of the etching steps in the Bosch sequence, Hirose et al.^8^ introduced a large scallop after a sequence of standard Bosch etching steps. The Bosch process is one of the methods most commonly used to create Si structures with high aspect ratios. This technique relies on repeated switching between isotropic etching and passivation to obtain a cavity with straight sidewalls. Combining standard Bosch processing with a purely isotropic etching process, Marty et al.^9^ reported the fabrication of multiple scallops along sidewalls and indicated its potential application in photonics, electronics, and microfluidics. Further optimizing the Bosch process, Chang et al. reported a correct 3-step Bosch process termed DREM, which can be used for patterning freestanding 3D silicon microstructures and nanostructures^10,11^. However, in these examples, the horizontal depth of the reported scallops is shallow and therefore limits their use for further patterning into sidewalls using corner lithography^12^. Corner lithography is based on conformal layer deposition and subsequent timed isotropic etching of the layer to leave a well-defined residue in concave corners, while on flat surfaces and convex corners, the layer is already removed^12^. Using this residue, various structures can be fabricated, for example nanorings^13^, octahedral features, and fractals^12,14^. The structures resulting from corner lithography have been applied, for example, in specialized AFM probes^12^, devices for cell trapping^15^, brain-on-a chip^16^, and gas permeation^17^.

In this paper, a multistep plasma etching process and corner lithography are combined to create a sidewall mask, which can be used for the fabrication of 3D structures repeated at defined depths in the bulk of Si wafers. Here, semicircular gaps are introduced at various depths using our multistep plasma process. These obtained semicircular gaps have a stronger curvature than the scallops created by the Bosch process, thus being suitable for corner lithography. To leave residues only inside these semicircular gaps, two types of corner lithography were investigated—one based on silicon nitride and another based on so-called digitally etched polysilicon. In addition, we demonstrated the potential use of this technique for patterning high aspect ratio 3D structures at the wafer scale by combining anisotropic (wet) with isotropic (dry) techniques at each semicircular gap.

## Results and discussion

### Theoretical calculation

**Fig. 1:**
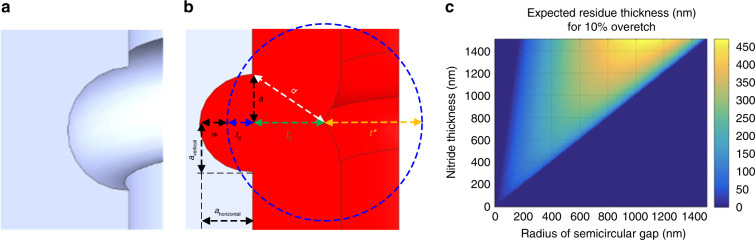
Illustrations of corner lithography and relationship between the semicircle radius, the thickness of deposited layer and the residue thickness. **a** Geometry of a semicircular gap. **b** Conformally deposited layer (red) and isotropic distance (blue) from the point on the layer that is closest to the semicircular gap. **c** Relationship between the semicircle radius, the thickness of the conformally deposited layer and the residue thickness. *a* is the radius of the semicircular gap, *w* is the residue thickness, *d* is the thickness of the deposited layer, and t* is the isotropic etching distance assuming 10% over-etching

Corner lithography is based on conformal deposition and subsequent well-timed isotropic etching to obtain a residue in a concave corner. In detail, the growth front in the conformal deposition process propagates faster at the surface with negative curvature (concave corner) when the film thickness exceeds the radius of curvature, while isotropic etching removes material with the same speed in all directions, as shown in Fig. 1. To determine the dimension of the circular gap, a model was constructed to demonstrate the relation between the dimension of the semicircular gap, thickness of the deposited layer and residue size. This model assumes that the gap etched into the Si has a perfect semicircular cross section and that the layer used for corner lithography is 10% over-etched (Fig. 1b). Details of the calculation are given in Supplementary Information section SI1. Figure 1c shows that generally for a thicker layer, a larger residue can be obtained. For a maximum deposition thickness of 1000 nm, a maximum residue thickness of 300 nm can be obtained for a gap radius of 650 nm. However, this model is only valid if the gap is etched in an ideally isotropic manner. If the profile of the gap is not ideal, *a*_horizontal_ = *a*_vertical_ − ∈, then ∈, which is the difference between the radius of the semicircular gap in the vertical and horizontal directions, has to be subtracted from the anticipated residue thickness. Therefore, if the gap has an obvious scallop-like characteristic (*a*_horizontal_ ≪ *a*_vertical_), the residues could be removed simultaneously from the gaps and the sidewall, which leads to corner lithography failure.

### Fabrication of high aspect ratio microholes with multiple semicircular gaps

**Fig. 2:**
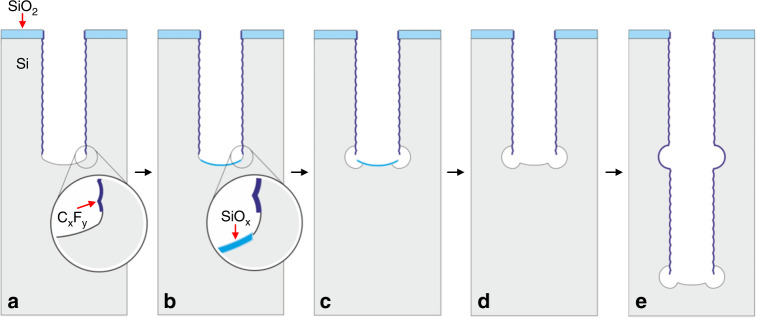
Fabrication process of a Si microhole with semicircular gaps in its sidewall using a multistep plasma etching process. **a** A high aspect ratio microhole etched in Si using a standard Bosch process; the process ended with an SF_6_ etching step. **b** Local plasma oxidation of Si was used to protect the bottom of the microhole. **c** Isotropic etching of Si using SF_6_ plasma to create a semicircular gap in the sidewall. **d** Etching of the remaining SiO_x_ layer at the bottom of the microhole using a CHF_3_/O_2_ plasma. **e** Repeating the multistep plasma etching process resulted in a high aspect ratio microhole with multiple semicircular gaps

Figure 2 shows the fabrication process of high aspect ratio microhole structures with semicircular gaps in the sidewall using the multistep plasma etching process that was developed for this purpose on an SPTS Pegasus system (SPTS, UK). Details of the fabrication process are given in the Materials and Methods section and Table 1. Briefly, a standard Bosch process with a standard scallop size was applied to etch high aspect ratio microholes. The Bosch process ended with a sulfur hexafluoride (SF_6_) plasma etch step. Therefore, the bottom surface of the last scallop in the microhole is not covered with fluorocarbon (C_x_F_y_) (Fig. 2a). Subsequently, a low-pressure oxygen (O_2_) plasma was applied to locally oxidize the bare Si at the microhole bottom surface (Fig. 2b). Thereafter, the formation of a semicircular gap was achieved by an isotropic etching process using an SF_6_ plasma (Fig. 1c). The plasma oxidized layer (SiO_x_) created at the bottom of the microhole was then removed by using a low-pressure plasma of CHF_3_ and O_2_ (Fig. 2d) with a capacitively coupled plasma (CCP) power of 100 W. By repeating these steps, i.e., the Si Bosch etching process, the local plasma oxidation of Si at the bottom, the isotropic etching of the semicircular gap using an SF_6_ plasma, and the removal of the SiO_x_ layer, a high aspect ratio microhole with repeated semicircular gaps in the sidewall can be produced (Fig. 2e).

**Table 1:** Multistep plasma process with corresponding times for creating three semicircular gaps at the sidewall of a microhole

Description	Repeating times	Multi-step plasma process	Duration (s)
1st DRIE (Bosch) of Si microholes	50	Deposition of C_4_F_8_	0.6
		Etching of Si by SF_6_	1.75
Fabricating of 1st semicircular gap	1	Local plasma oxidation of Si	2
		Isotropic sidewall etching by SF_6_	5.2
		Etching of SiO_x_ layer by CHF_3_+O_2_	60
2nd DRIE (Bosch) of Si microholes	50	Deposition of C_4_F_8_	0.6
		Etching of Si by SF_6_	1.75
Fabricating of 2nd semicircular gap	1	local plasma oxidation of Si	2
		Isotropic sidewall etching by SF_6_	5.6
		Etching of SiO_x_ layer by CHF_3_+O_2_	60
DRIE (Bosch) of Si microholes	50	Deposition of C_4_F_8_	0.6
		Etching of Si by SF_6_	1.75
Fabrication of 3rd semicircular gap	1	Local plasma oxidation of Si	2
		Isotropic sidewall etching by SF_6_	6.0
		Etching of SiO_x_ layer by CHF_3_+O_2_	60
3rd DRIE (Bosch) of Si microholes	50	Deposition of C_4_F_8_	0.6
		Etching of Si by SF_6_	1.75

**Fig. 3:**
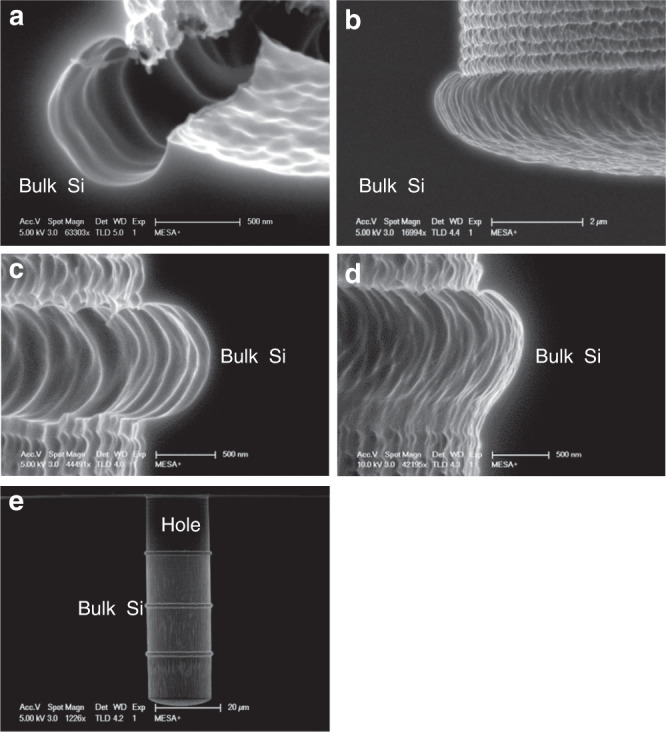
Cross-sectional HR-SEM images of **a** a semicircular gap after the Bosch process, plasma oxidation of Si and isotropic plasma etching of Si; **b** a structure after the Bosch process and isotropic plasma etching of Si; **c** the semicircular gap in **a** after the second Bosch process; **d** the structure in **b** after the second Bosch process without the plasma oxidation step, having an ear shape; and **e** a microhole with three repeated semicircular gaps

Figure 3a shows a cross-sectional HR-SEM image of a semicircular gap of 500 nm after performing the multistep plasma process sequence: the Bosch process, the local plasma oxygen of Si and the isotropic etching of the sidewall. Figure 3b shows the result after only performing the Bosch process and the isotropic etching of the bottom of the high aspect ratio microhole. Comparing Fig. 3a with b, we observed that the low-pressure plasma oxidation step only oxidized the bare Si at the horizontal part of the bottom of the microhole. The defined sidewall gap of bare Si at the bottom of the microhole enhanced the isotropic etching of Si, thereby creating a semicircular gap of approximately 500 nm.

Without the oxygen plasma step of Si, the bottom of the microhole and the sidewall were both etched during the isotropic etching step. Due to a higher flux of ions and radicals at the center of the bottom of the microhole, the etching rate is higher there, which results in an “ear shaped” profile (see Fig. 3d).

By applying the oxygen plasma step, the bottom surface of the Si microhole was protected. As a result, the isotropic etching process only contributed laterally from the sidewall, which effectively reduced the influence of the nonuniform etching in the vertical direction. Figure 3c, d show the resulting structure after further etching of the straight part of the sidewall. From Fig. 3c, we can observe that a nearly perfect semicircular Si gap with ~1.14 μm vertical diameter and 620 nm horizontal radius was obtained, which is suitable for subsequent corner lithography. Without the directional oxygen plasma step, the resulting structure, as shown in Fig. 3d, is ear shaped. This ear-shaped feature had a vertical diameter of approximately 1.29 μm and a horizontal radius of 420 nm, which can result in complete removal of the residues during wet etching of the deposited film in a later step, thus leading to corner lithography failure.

### Sidewall patterning of 3D structures using corner lithography

**Fig. 4:**
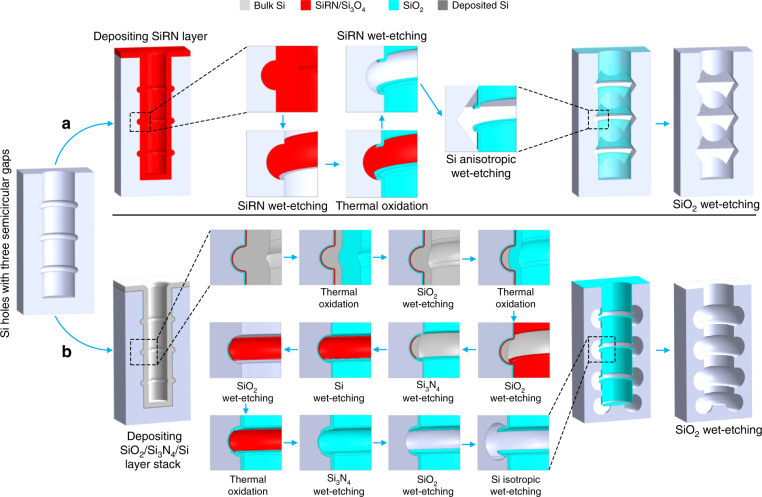
Schematic diagram of sidewall patterning of repeated 3D structures using **a** silicon nitride-based corner lithography and **b** digital etching-based corner lithography

Figure 4 shows the schematic diagram of sidewall patterning of repeated octahedra using silicon nitride-based corner lithography (Fig. 4a) and of repeated donuts using alternative digital etching-based corner lithography (Fig. 4b). Details of the fabrication process are given in the “Materials and methods” section. It is worth mentioning that the final repeated structures (octahedra or donuts) depend on the etching method that is applied to the bulk Si. Therefore, it is possible to fabricate repeated donuts via silicon nitride-based corner lithography and repeated octahedra via digitally etched polysilicon corner lithography.

#### Silicon nitride-based corner lithography

**Fig. 5:**
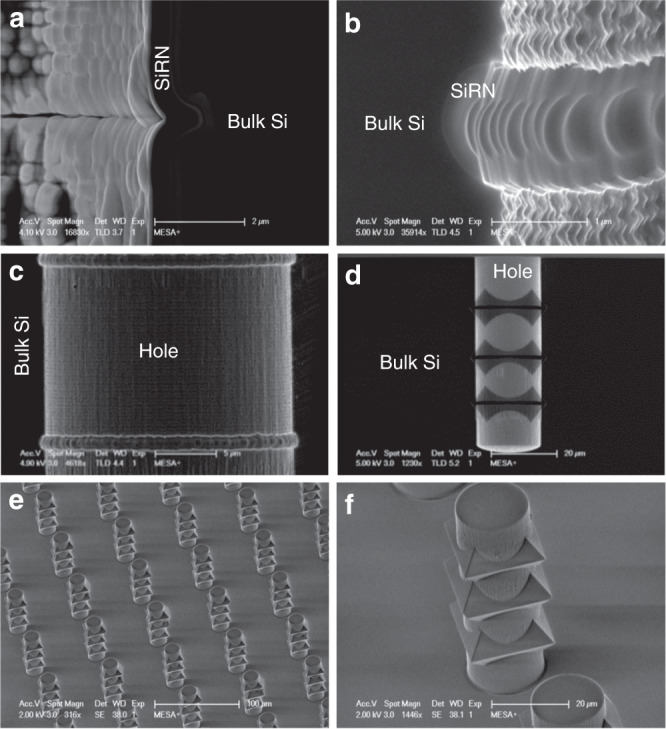
Cross-sectional HR-SEM images of **a** a semicircular gap filled with a thick SiRN layer of approximately 1 μm, **b** residue left only inside the semicircular gap after etching in the modified phosphoric acid solution, **c** straight sidewall of a microhole after etching in the modified phosphoric acid, **d** a microhole with repeated octahedra, and **e**, **f** released inverted structures made inside the Si wafer

Figure 5a shows a cross-sectional HR-SEM image of a semicircular gap filled with a thick SiRN layer of approximately 1 μm. The SiRN layer was conformally deposited via LPCVD. The observed roughness on the outermost surface of the SiRN layer was probably caused by the original roughness of the Si sidewall. After isotropic etching of the sample in a modified (see the “Materials and methods” section) 85% H_3_PO_4_ solution at 180 °C for 7 h, a thin SiRN layer remained only inside the semicircular gap (Fig. 5b, c). We attribute this to the fact that a thicker SiRN layer was deposited on the semicircular gaps compared to that deposited on the straight part of the sidewall. Isotropic etching of SiRN for a sufficient time resulted in a residue of SiRN only inside each semicircular gap.

To achieve precise control in the etching of SiRN in all three semicircular gaps through the depth of the high aspect ratio structures, an amount of ~1.6 mg SiRN was dissolved into a 3.5 L etching solution (85% H_3_PO_4_) to reduce the etching rate of SiRN from ~6.9 to ~3.3 nm min^−1^. Without such modification of the etching solution, the etching rate of SiRN gradually decreased with the increasing depth of the high aspect ratio structure due to the limited mass transport. This could result in a finite layer or residue left on the sidewalls deeper inside the high aspect ratio structures. Moreover, the SiRN inside the first semicircular gap could be completely removed before the unexpected layer of SiRN on the sidewall near the third semicircular gap was properly eliminated, which can result in an imperfect SiRN mask and ultimately lead to the formation of an undesired 3D structure after corner lithography.

After corner lithography of the SiRN layer inside the three Si semicircular gaps, a LOCOS process was conducted at 1050 °C for 30 min to form a sidewall SiO_2_ mask, and subsequently, the remaining SiRN was stripped in a hot 85% H_3_PO_4_ solution at 180 °C. Using this SiO_2_ mask, three octahedra at the location of the three semicircular gaps were fabricated by immersing the sample in a 25 wt% TMAH solution at 70 °C for 45 min (Fig. 5d). The shapes were inverted by removing the SiO_2_ mask, conformally depositing a SiRN layer, anodically bonding to a glass wafer and subsequently etching the bulk Si substrate, so 3D structures consisting of repeated octahedra made of SiRN were fabricated over large areas (Fig. 5e, f).

#### Digitally etched polysilicon corner lithography

The success of silicon nitride-based corner lithography depends on the uniformity of nitride etching. Despite the modification of the etching solution, etch speed differences were found between the top and bottom of high aspect ratio structures (Supplementary SI4). For higher aspect ratio structures, the topmost residues are completely etched before the sidewalls at the bottom are free of nitride. To make the process less dependent on the etching uniformity, an alternative approach was applied: digital etching-based polysilicon corner lithography.

**Fig. 6:**
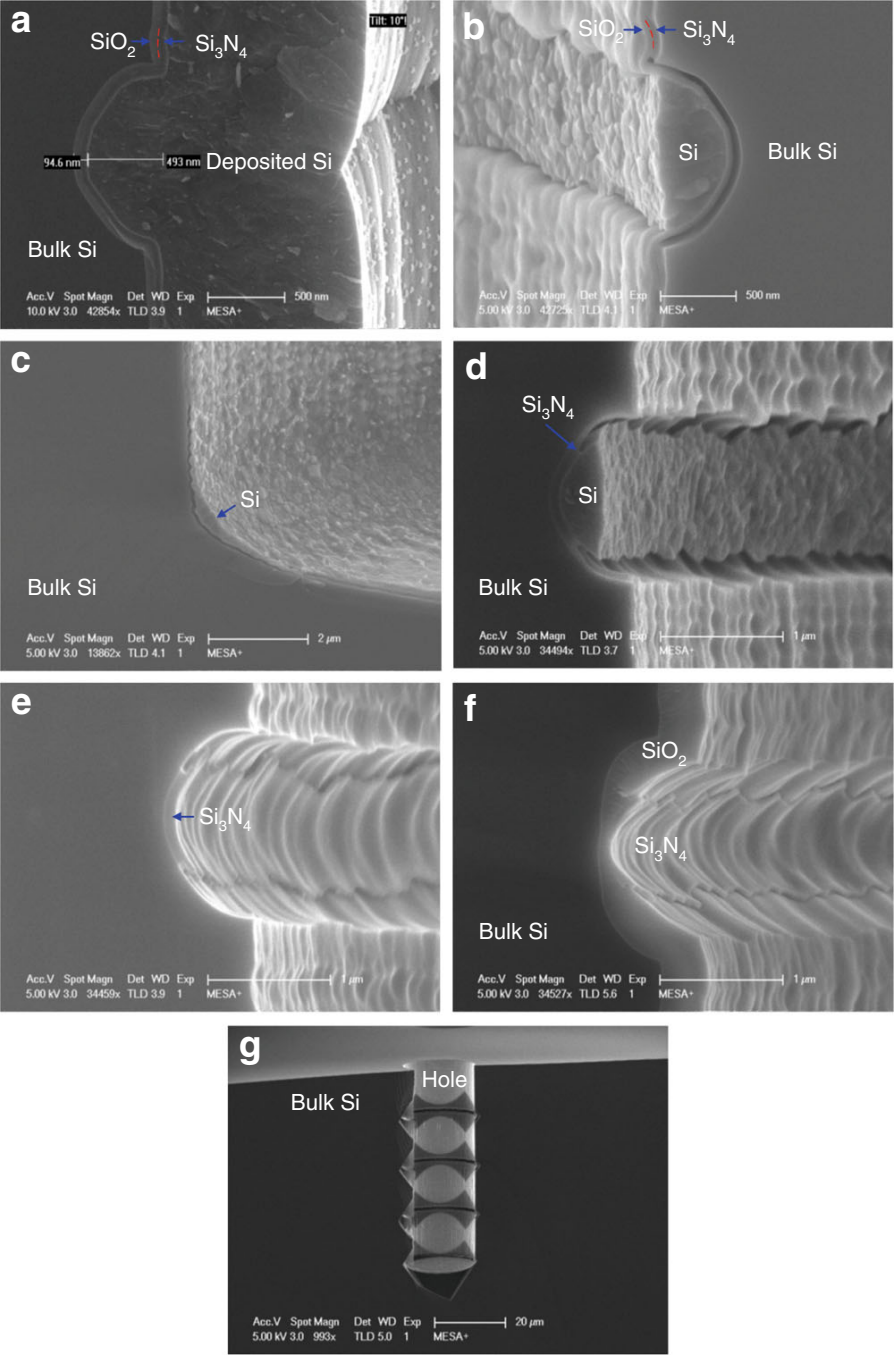
Cross-sectional HR-SEM images of **a** a semicircular gap filled with stacked layers: (from the outermost surface) a polysilicon layer of ~1.3 μm, a Si_3_N_4_ layer of ~50 nm, and a SiO_2_ layer of ~50 nm; **b** this semicircular gap after digital etching; **c** the bottom corner of the microhole after digital etching; **d** the structure after retraction of the stoichiometric nitride layer along the edge of the polysilicon residue; **e** the structure after the retraction of the thin oxide layer in 1% HF solution; **f** profile of the semicircular gap after LOCOS; **g** the structure with repeated octahedra resulting from the combination of corner and edge lithography

Figure 6a shows a cross-sectional HR-SEM image of a semicircular gap filled with conformally deposited layers from the outermost surface: a polysilicon layer of ~1.3 μm, a Si_3_N_4_ layer of ~50 nm, and a SiO_2_ layer of ~50 nm. By applying a digital etching process (Supporting Information Table S12), the polysilicon layer on the straight part of the sidewall of the high aspect ratio structures was etched completely, whereas polysilicon residues approximately 450-nm thick remained inside the semicircular gaps (Fig. 6b). It is worth mentioning that an insufficient duration of the last oxidation step of the digital etching process could lead to a residual layer of polysilicon (~370 nm) at the bottom corner of the Si high aspect ratio structures as well (Fig. 6c). We attribute this to the rounding effect of the thermal oxidation process^18,19^.

The Si_3_N_4_ layer was then retracted along the edge of the remaining polysilicon inside the Si semicircular gap (Fig. 6d), thus providing precise control of the Si_3_N_4_ etching underneath the polysilicon layer. The retraction through the edge further widens the over-etching window, as the outer surface of the remaining nitride layer is protected by the residue, and the etchant can only remove the material through the edge of the residue. This over-etching would lift off small unwanted polysilicon residues at the sidewall and therefore avoid imperfections in the final out-of-plane mask to a large extent. After the retraction of the Si_3_N_4_ layer, the polysilicon layer was completely etched in a 25% TMAH solution at 70 °C for 45 s. Subsequently, the exposed SiO_2_ layer was etched in a 1% HF solution for 22 min (Fig. 6e). Thereafter, a LOCOS process was conducted at 1050 °C to form a SiO_2_ layer of ~300 nm at the sidewall of the high aspect ratio Si microhole. Figure 6f clearly shows that inside the semicircular gap, the SiO_2_ layer under the edge of the Si_3_N_4_ layer was much thicker than that underneath the center of the Si_3_N_4_ layer. We attribute this to the fact that oxygen more easily diffuses under the edge of the Si_3_N_4_ layer than through the complete Si_3_N_4_ layer during the LOCOS process. After removing the Si_3_N_4_ layer in a hot H_3_PO_4_ solution at 180 °C, a thin silicon oxide layer remained inside the semicircular holes, while a thicker layer was grown on the vertical sidewalls. The sample was then etched in a 1% HF solution for 36 min to remove the thinner SiO_2_ layer inside the Si semicircular gaps, thereby leaving a masking layer of SiO_2_ on the vertical sidewalls. Using this masking layer, repeated octahedra at the location of three Si semicircular gaps were fabricated (Fig. 6g). Moreover, an additional octahedron was also formed at the bottom of the Si high aspect ratio microholes due to the polysilicon residues resulting from the rounding effect of the thermal oxidation steps used in the digital etching process.

**Fig. 7:**
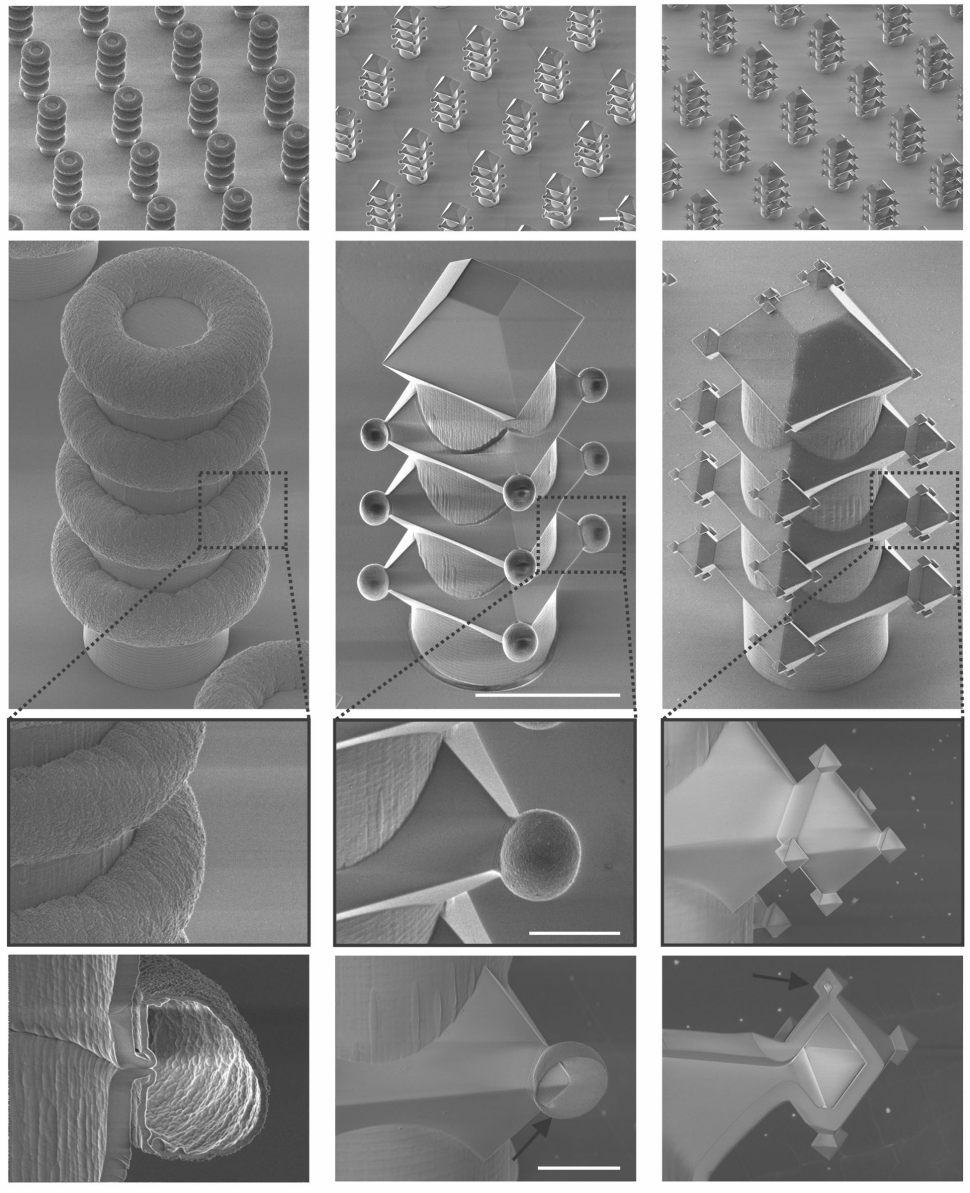
Cross-sectional HR-SEM images of various 3D structures fabricated by using different combinations of isotropic etching with anisotropic etching. Scale bars represent 20 µm (first and second rows) and 5 µm (third and fourth rows)

Using the sidewall SiO_2_ masking layer, various complicated structures, such as repeated donuts, fractals, and balls, could be fabricated by applying different combinations of isotropic etching (XeF_2_ etching) and anisotropic etching (TMAH etching), as shown in Fig. 7. In this way, the density of features can be increased at will, as they self-align in both horizontal and vertical directions.

## Conclusions and outlook

In summary, we report a fabrication method for wafer-scale sidewall patterning that is repeatable in depth and combines a multistep plasma etching process with corner lithography. The presented multistep plasma etching consists of a standard Bosch etching process, a low-pressure plasma oxidation process of Si, a high-pressure isotropic SF_6_ etching process, and the use of low-pressure CHF_3_/O_2_ plasma to remove the remaining oxidized Si layer. The embedding of the plasma oxidation step makes it possible to control the shape and uniformity of the circular gap at the sidewall of the high aspect ratio microholes. The multistep process can be easily repeated, thereby creating semicircular gaps in the sidewalls with various high aspect ratio structures etched in Si. These semicircular gaps were used as concave corners for corner lithography, leaving residues only inside these semicircular gaps and thereby forming a sidewall mask. Using this mask, repeated donuts and fractals were successfully fabricated via further etching processes, which demonstrate the potential of this technology for the fabrication of high-density and high aspect ratio repeated 3D structures inside bulk Si. While the current demonstrated structures are based on hole diameters of several micrometers, it is expected that the method is applicable on the submicron scale as well. High-density, tall 3D structures could find applications in fields ranging from electronics^20^ and energy storage^21^ to optics^22^ and microfluidics^23^.

One ongoing research line focuses on the fabrication of double cross-flow 3D-micromixers using the reported fabrication method. Arrays of vertical, perforated tubes will be integrated in a microfluidic device such that the space between the tubes constitutes a (horizontal) microfluidic channel. A first step towards such a device, consisting of a 3D perforated fractal, has been used for gas permeation experiments^17^. We believe that the permeation efficiency or the mixing efficiency can be further increased using our cross-flow 3D-micromixers integrated with repeated fractals, which can be fabricated by combining our multi-step plasma etching process with corner lithography and anisotropic wet etching. The double cross-flow configuration is a promising candidate to reduce clogging issues typical for dead-end configurations.

## Materials and methods

### Patterning SiO_2_ microholes on Si substrates as the masking layer

First, 100-mm diameter (100) oriented Si wafers (525-μm thick, Okmetic, Finland) with a thermal silicon dioxide (SiO_2_) layer of approximately 1 μm were used as substrates for all fabrication processes. This thermal oxide layer was formed by wet oxidation in a high-temperature tube furnace (Model 287, TEMPRESS, Netherlands) at 1150 °C for 2 h and 40 min (Table S1). Periodic microholes (20 μm diameter, 60 μm pitch) were patterned in photoresist (OiR 907–17 Fujifilm, Japan) on the surface of these oxidized Si-wafers by using photolithography (EVG620 mask aligner, EV Group, Austria) and subsequently used as a mask for patterning microholes into the thermal oxide layer by reactive ion etching (AMS100DE, Adixen, France). Details of this etching process are given in Table S2. These patterned microholes in the SiO_2_ layer were used as a hard mask to allow for deep etching of the Si substrate using a multistep plasma etching process.

### Fabrication of high aspect ratio microholes with semicircular gaps

A standard Bosch process with a standard scallop size was applied to etch high aspect ratio microholes. The Bosch process ended with a sulfur hexafluoride (SF_6_) plasma etching cycle. (Fig. 2a). Subsequently, low-pressure oxygen plasma was applied to locally oxidize the bare Si at the bottom surface of the high aspect ratio microhole (Fig. 2b). Thereafter, the formation of a semicircular gap was achieved by an isotropic etching process using SF_6_ plasma (Fig. 2c). The plasma oxidized layer (SiO_x_) created at the bottom of the microhole was then removed by using a low-pressure plasma of CHF_3_ and O_2_ (Fig. 2d) with a CCP power of 100 W. By repeating these steps, i.e., the Si Bosch etching process, local plasma oxidation of Si at the bottom, isotropic etching of the semicircular gap using an SF_6_ plasma, and removal of the SiO_x_ layer, high aspect ratio microholes with repeated semicircular gaps in the sidewall were fabricated (Fig. 2e). Details of the multistep plasma process are given in Tables S3–S6. The multistep process for the fabrication of Si microholes with three semicircular gaps is summarized in Table 1.

### Silicon nitride-based corner lithography

A thick silicon-rich nitride (SiRN) layer of approximately 1 μm was conformally deposited over a Si microhole with three semicircular gaps (Fig. 4a) by low-pressure chemical vapor deposition (LPCVD) in a high-temperature tube furnace (TC6304, TEMPRESS, Netherlands), as shown in Table S7, and subsequently etched isotropically in an 85 wt% phosphoric acid (H_3_PO_4_) solution at 180 °C for 7 h. Before the etching process, the solution was modified by dissolving ~1.6 mg of SiRN into a 3.5 L etching solution to slightly saturate the solution and thereby decrease the etching rate from ~6.9 nm min^−1^ (fresh solution) to ~3.3 nm min^−1^. The etching rate of the phosphoric acid was monitored by simultaneously etching dummy wafers. The residues of the SiRN layer inside the semicircular gaps after SiRN etching were used as a mask to perform the local oxidation of Si (LOCOS) process at 1050 °C for 30 min (Table S8). Thereafter, the remaining SiRN layer was stripped in the hot H_3_PO_4_ solution, resulting in a sidewall layer of SiO_2_. Further etching in a 25 wt% tetramethylammonium hydroxide (TMAH) solution at 70 °C for 45 min resulted in three octahedra at the location of the three semicircular gaps.

### Digital etching-based polysilicon corner lithography

A Si microhole with three semicircular gaps was dry oxidized at 1050 °C for 25 min (TC6304, TEMPRESS, Netherlands, Table S9), followed by an oxide stripping process in a 50% HF solution for 30 s to smooth the sidewall of the Si microholes (Fig. 4b). The principle of sidewall smoothing can be found in Supplementary SI5. Subsequently, another SiO_2_ layer of approximately 50 nm was formed over the structure surface by using dry oxidation at 1050 °C for 25 min. Thereafter, an ~50-nm thick stoichiometric silicon nitride (Si_3_N_4_) layer and an ~1.3-μm polysilicon layer were conformally deposited using LCPVD processes. The process parameters are given in the Tables S10, S11. Subsequently, a digital etching process was applied to leave polysilicon residues only inside the semicircles. Each digital etching step consists of a thermal oxidation step at 1050 °C and a selective oxide stripping step in a 50% HF solution. Details of all the digital etching steps used are given in Table S12. By using these steps, the size and shape of the polysilicon residues were controlled by the oxidation process instead of the wet chemical etching process. The Si_3_N_4_ layer was etched in a hot H_3_PO_4_ solution with an etching rate of 3.8 nm min^−1^, leaving a remaining Si_3_N_4_ layer only underneath the polysilicon residues. This edge lithography step^24,25^ was used to obtain precise retraction of an etched layer underneath a masking layer. The polysilicon residues were completely etched in a 25% TMAH solution at 70 °C for 45 min, whereas the SiO_2_ layer served as a protecting layer for the bulk Si substrate. Subsequently, this SiO_2_ layer was also retracted in a 1 wt% HF solution at room temperature for 22 min with an etching rate of 4.6 nm min^−1^, followed by a LOCOS process at 1050 °C to form a SiO_2_ layer of ~300 nm at the sidewall of the Si high aspect ratio microholes. Thereafter, an etching step in a 1% HF solution for 45 s was performed to remove the oxidized surface of the remaining Si_3_N_4_ layer before completely stripping this Si_3_N_4_ layer in a hot H_3_PO_4_ solution. The sample was then etched in a 1% HF solution for 36 min to thin down the SiO_2_ layer so that the thinner SiO_2_ layer inside the large scallops was completely removed while leaving some of the thicker layer on the vertical sidewalls as a masking layer.

### Fabrication and visualization of repeated 3D structures

Various repeated 3D structures were fabricated by using the sidewall mask, as shown in Figs. 5e, f, 7. To obtain the repeated octahedral structures shown in Fig. 5e, f, a SiRN layer of approximately 750 nm was deposited conformally on structures that are shown in Fig. 5d using the LPCVD process (Table S7). Thereafter, the samples with 3D structures were anodically bonded to glass wafers. The principle for the anodic bonding of SiRN to glass was reported elsewhere^26,27^. For a high bonding strength, samples with a SiRN layer were wet-oxidized at 1100 °C for 30 min (Model 287, TEMPRESS, Netherlands, Table SI), and the glass wafers were cleaned by immersion into a piranha solution (95 °C, H_2_SO_4_:30 wt% H_2_O_2_ = 3:1). The anodic bonding was performed using a voltage of 1000 V at a temperature of 450 °C for 30 min with a point contact on the glass. Subsequently, the samples were fractured to create a cross section through the 3D structures, and potassium hydroxide etching (KOH, 25 wt%, 75 °C, etch rate of ~1 μm min^−1^) was then applied to remove the bulk Si.

To obtain repeated donuts, as shown in the first column of Fig. 7, ten cycles of XeF_2_ etching (Xacitix, Table S13) were conducted for the structures shown in Fig. 6g. Thereafter, a SiRN layer was deposited, and the samples with the 3D structures were anodically bonded to the glass wafers, followed by etching the remaining Si in the KOH solution.

Oxide-only corner lithography^28^ was applied to fabricate the structures shown in the second column of Fig. 7. The structures shown in Figs. 5d or 6g were dry oxidized at 1100 °C for 95 min (TC6304, TEMPRESS, Netherlands, Table S9), thus resulting in another SiO_2_ layer of approximately 163 nm. Subsequently, these samples were immersed in a 1 wt% HF solution for 20 min and 50 s (etch rate of ~4.3 nm min^−1^ at room temperature). As a result, the apertures of the repeated octahedra were opened, as shown in Figs. 5d or 6g. Thereafter, 10 cycles of XeF_2_ etching (Xacitix, Table S13) were conducted, resulting in round features at the apertures of the repeated octahedra. Finally, these samples were covered with a SiRN layer and anodically bonded to glass wafers, followed by etching the remaining Si in the KOH solution.

For the structures shown in the third column of Fig. 7, samples with the structures shown in the second column of Fig. 7 were immersed in a TMAH solution (25 wt%, 70 °C). As a result, the second generation of repeated octahedra was fabricated. Thereafter, dry oxidation at 1100 °C was conducted for 95 min (TC6304, TEMPRESS, Netherlands, Table S9) to form another SiO_2_ layer of approximately 163 nm, followed by etching in the 1 wt% HF solution (etch rate of ~4.3 nm min^−1^ at room temperature) for 20 min and 50 s to open the apertures of the second-order octahedra. Subsequently, TMAH etching (25 wt%, 70 °C) was applied for 70 min to create the third generation of octahedra. The resulting structures were then immersed in a 50% HF solution for 30 s to remove the SiO_2_ masking layer. Finally, a SiRN layer was deposited, and the sample was then anodically bonded to glass wafers, followed by etching in the KOH solution to remove the bulk Si.

## Supplementary information

Supplemental material
