# Supplementary material for: Wafer-scale 3D shaping of high aspect ratio structures by multistep plasma etching and corner lithography
Source: Microsyst Nanoeng. 2020 Mar 23;6:25. doi: 10.1038/s41378-020-0134-6 (PMC8433478; doi:10.1038/s41378-020-0134-6)
Supplement: Supplementary file 1 — Supplemental material [file 41378_2020_134_MOESM1_ESM.pdf]

Supplementary Information

Wafer-scale 3D shaping of high aspect ratio structures by multi-step plasma etching and corner lithography

Shu Ni[1,2], Erwin J. W. Berenschot[1], Pieter Westerik[1], Meint J. de Boer[3], Rene Wolf[3], Hai Le-The[4,5], Han J. G. E. Gardeniers[1] and Niels R. Tas[1]

[1]Mesoscale Chemical System Group, MESA+ Institute, University of Twente, 7522 NB Enschede, The Netherlands
[2]Inorganic Materials Science Group, MESA+ Institute, University of Twente, 7522 NB Enschede, The Netherlands
[3]NanoLab Cleanroom, MESA+ Institute, University of Twente, 7522 NB Enschede, The Netherlands
[4]Physics of Fluids Group, MESA+ Institute & Max Planck Center for Complex Fluid Dynamics, University of Twente, Enschede 7522 NB, The Netherlands
[5]BIOS Lab-on-a-Chip Group, MESA+ Institute & Max Planck Center for Complex Fluid Dynamics, University of Twente, Enschede 7522 NB, The Netherlands

SI1. Details of the theoretical calculation

To determine the dimension of the circular gap, a model is constructed to demonstrate the relation between the dimension of the semicircular gap, the thickness of the deposited layer and the residue size. This model assumes that the gap etched into the silicon has a perfect circular cross section, and that the layer used for corner lithography is 10% over-etched, as shown in Fig. 1a and 1b in the main article.

Assuming that the silicon nitride etching is ideally isotropic, we have

$$t^{*} = t_2 + t_3 \tag{S1}$$

From the geometry point of view, we can conclude that

$$t_2^2 = d^2 + a^2 \tag{S2}$$

$$w = a - t_3 \tag{S3}$$

Therefore, we have:

$$w = a + t_2 - t^{*} \tag{S4}$$

Combine these expressions, we have:

$$w = a + \sqrt{d^2 - a^2} - t^{*} \tag{S5}$$

For determining the relation between the size of the circular gap and the layer thickness of SiRN, it was assumed that 10% over-etching (k) would be enough to ensure that residues are only present in the big scallops thus:

$$t^{*} = k \cdot d \tag{S6}$$

$$k = 1.1 \tag{S7}$$

Therefore, we can obtain that:

$$w = a + \sqrt{d^2 - a^2} - 1.1d \tag{S8}$$

Based on this final expression, we obtain the relation between the radius of the circular gap, the thickness of the silicon nitride and the residue size, as shown in Fig. 1c in the main article.

SI2. Details of the etching and deposition parameters

Table S1. Recipe for growing 1 μm silicon dioxide on Si

Parameters	Value
Standby temperature (°C)	700
Temperature range (°C)	700 ~ 1150
Ramp (°C min^{-1})	10
Oxygen flow (L min^{-1})	4

Table S2. Recipe for etching the silicon dioxide with microholes

Parameters	Value
Gas	CHF_4/C_4F_8; He
Flow (sccm)	CHF_4:15; C_4F_8: 20; He: 150
Time (s)	150
Pressure (mbar)	8.5×10^{-13}
ICP (W)	280
CCP(W)	350

Table S3. Recipe for the Bosch process

Parameters	Etch	Deposition
Gas	SF_6	C_4F_8
Flow (sccm)	275	150
Priority	2	1
Time (s)	1.75	0.6
Pressure (mTorr)	26	20
ICP (W)	2200	2000
CCP (W)	20 – 30	0
LF pulses	20%	

Table S4. Recipe for local plasma oxidation of Si

Parameters	Value
Gas	O_2
Flow (sccm)	200
Pressure (mTorr)	5
ICP (W)	2500
CCP (W)	100

Table S5. Recipe for the isotropic sidewall etching of semicircular gaps

Parameters	Value
Gas	SF_6
Flow (sccm)	275
Pressure (mTorr)	26
ICP (watts)	2200
CCP (watts)	0

Table S6. Recipe for the etching of local oxide layer

Parameters	Value
Gas	$CHF_3 + O_2$
Flow (sccm)	CHF_3: 200; O_2: 35
Pressure (mTorr)	26
Time (s)	60
ICP (W)	2500
CCP (W)	100
LF pulse	20%

Table S7. Recipe for the LPCVD of silicon rich nitride

Parameters	Value
Gas	$SiH_2Cl_2 + NH_3 + N_2$
Flow (sccm)	SiH_2Cl_2: 77.5; NH_3: 20; N_2: 250
Pressure (mTorr)	150
Temperature (^oC)	850

Table S8. Recipe for LOCOS

Parameters	Value
Gas	$N_2 + H_2O$
Flow (sccm)	16.722
Temperature (^oC)	1050

Table S9. Recipe for dry oxidation

Parameters	Value
Gas	O_2
Flow (sccm)	5000
Temperature (°C)	1050
Ramp (°C min^{-1})	10

Table S10. Recipe for the LPCVD of polysilicon

Parameters	Value
Gas	SiH_4 + N_2
Flow (sccm)	SiH_4: 250; N_2:250
Temperature (°C)	590
Pressure (mTorr)	250

Table S11. Recipe for the LPCVD of stoichiometric silicon nitride

Parameters	Value
Gas	SiH_2Cl_2 + NH_3 + N_2
Flow (sccm)	SiH_2Cl_2: 25; NH_3: 250; N_2: 250
Pressure (mTorr)	150
Temperature (°C)	750

Table S12. Detailed information of digital etching of polysilicon

Steps	Treatment	Thickness of resulting oxide at (111) plane
1	Dry oxidation of 240 min	910 nm
2	Stripping in 50% HF for 45 s	0 nm
3	Dry oxidation for 360 min	1088 nm
4	Stripping in 50% HF for 60 s	0 nm
5	Dry oxidation for 360 min	1088 nm
6	Stripping in 50% HF for 60 s	0 nm
7	Dry oxidation for 20 min	316 nm
8	Stripping in 50% HF for 20 s	0 nm

*Thickness of the resulting oxide at (111) plane was monitored by (111) silicon dummy wafers

Table S13. Recipe for XeF_2 etching

Parameters	Value
Gas	XeF_2
Etching time (s) / cycle	30
Temperature (°C)	35
Pressure (mTorr)	3000

SI3. Multi-step plasma etching for structures with openings of different sizes

To demonstrate the versatility of our technique, wafers with microholes of 5 μm and 10 μm in diameter were used for the multi-step plasma etching. Fig. S1a and S1c show that high aspect ratio structures with semicircular gaps were fabricated inside these holes. The horizontal radius and vertical diameter were approximately 297 nm and 538 nm for the 5 μm dimeter holes (Fig. S1b), and 446 nm and 866 nm for the 10 μm diameter holes (Fig. S1d).

For comparison, we have also performed the process reported by Hirose *et al*[1]. In short, an etching step of the standard Bosch process was increased to 5.2 s to create a gap at the sidewall. As shown in Fig. S2a, there was no obvious gap or a bigger scallop but a peculiar depletion region in the hole, which has a top opening of 5 μm in diameter. A slightly larger scallop, which has a horizontal radius of 104 nm and a vertical diameter of 384 nm, was formed in the hole, which has a top opening of 10 μm in diameter, as shown in Fig. S2b. A gap (big scallop) was observed in the hole, which has a top opening of 20 μm in diameter (Fig. S2c). The horizontal radius and the vertical diameter of this gap were approximately 292 nm, and 892 nm, respectively.

It can be concluded from Fig. S1 and Fig. S2 that our multi-step plasma etching can be used to create semicircular gaps inside the high aspect ratio structures, which have top openings of different diameters, compared to the method reported by Hirose *et al*[1]. Moreover, the resulting semicircular gaps were also suitable for corner lithography. The process reported by Hirose *et al.*[1] is highly dependent on the size of the top openings, and it can only be used to

create gaps for structures with large openings (>20 μm). Furthermore, the resulting gaps showed a "ear-shaped" profile, which could result in simultaneous removal of the deposited layer at the straight part of the sidewall and the gap, and thereby lead to the failure of corner lithography, as shown in Fig. S2d.

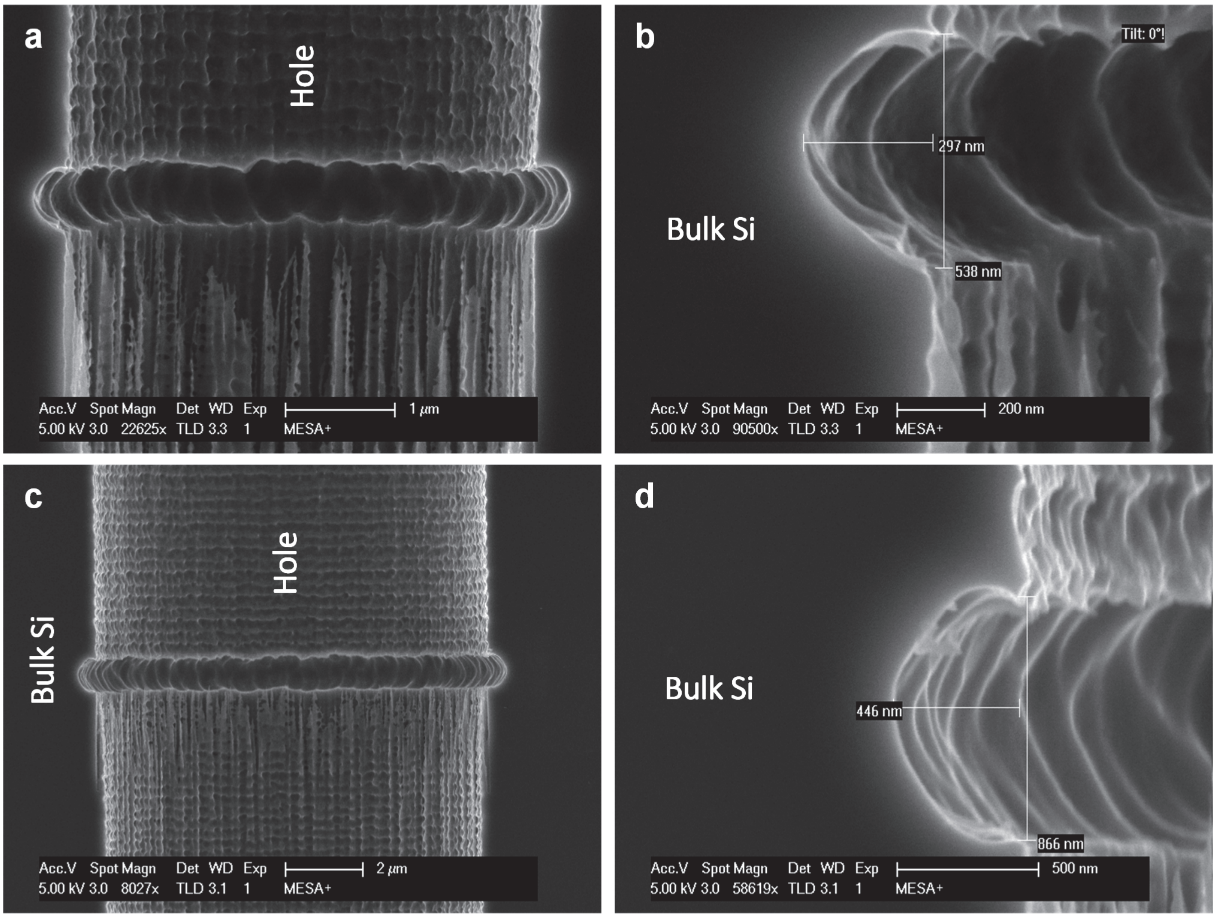

Fig. S1 Cross-sectional HR-SEM images of **a**,**b**) a semicircular gap inside a hole of 5 μm in diameter fabricated by multi-step plasma etching, and **c**,**d**) a semicircular gap inside a hole of 10 μm in diameter by multi-step plasma etching

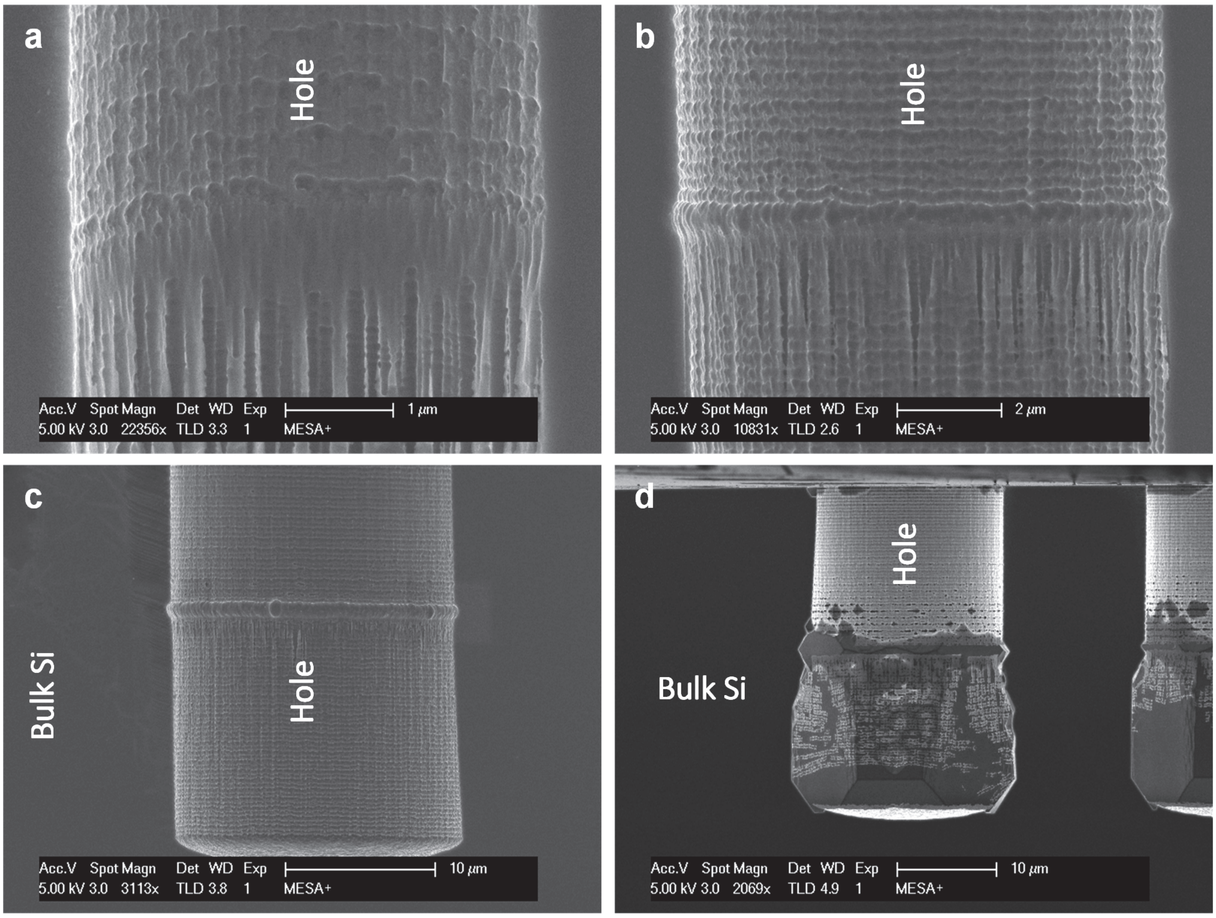

Fig. S2 Cross-sectional HR-SEM images of **a**) a structure with a top opening of 5 μm in diameter resulting from the process reported by Hirose *et al*[1], **b**) a structure with a top opening of 10 μm in diameter resulting from the process reported by Hirose *et al*[1], **c**) a structure with a top opening of 20 μm in diameter resulting from the process reported by Hirose *et al*[1], and **d**) a structure after performing corner lithography on the structure shown in Fig. S2c.

SI4. Conditions for uniform etching of silicon rich nitride

For silicon-nitride-based corner lithography, an uniform silicon nitride (SiRN) etching profile can only be obtained if the following two conditions are fulfilled: the etching of SiRN in phosphoric acid (H_3PO_4) is completed in one run, and the H_3PO_4 solution (85 wt% at 180°C) is pre-treated by dissolving SIRN so that the etch rate is lower than 3.5 nm min^{-1}

When the etching is performed in multiple runs, a peculiar "powder-like" material can be formed at the sidewall of the high aspect ratio microholes, as shown in Fig. S3. The "powder-

like” material may be a locally accumulated etching by-product: hydrated silicon oxide. Although the precise formation mechanism is unknown, we managed to remove this “powder-like” material by immersing the sample in 1% HF solution at room temperature for 10 min.

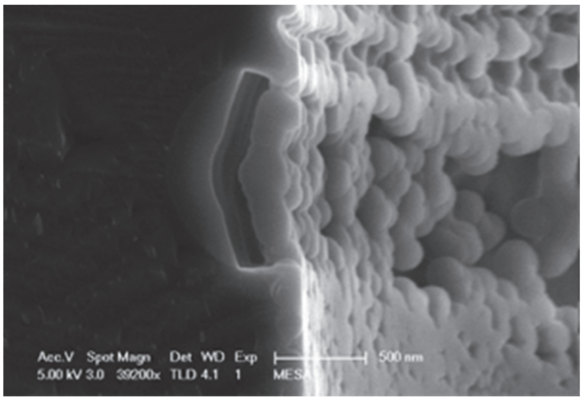

Fig. S3 “Powder-like” materials at the sidewall of a high aspect ratio microhole after the second run of etching in phosphoric acids.

Fig. S4 shows the SIRN etching profile after processing in the H_3PO_4 (85 wt% at 180ºC) solution with an etching rate of approximately 4.47 nm min^{-1}. From Fig. S4a and S4b, we can see that a profile transition occurred at the straight part of the sidewall, and the layer above this transition region was completely stripped but the part below it was covered with a thin layer. From Fig. S4c-e, we can see that the residue thicknesses at the semicircular gaps were 392 nm, 503 nm and 540 nm, respectively, which increased deeper inside the hole.

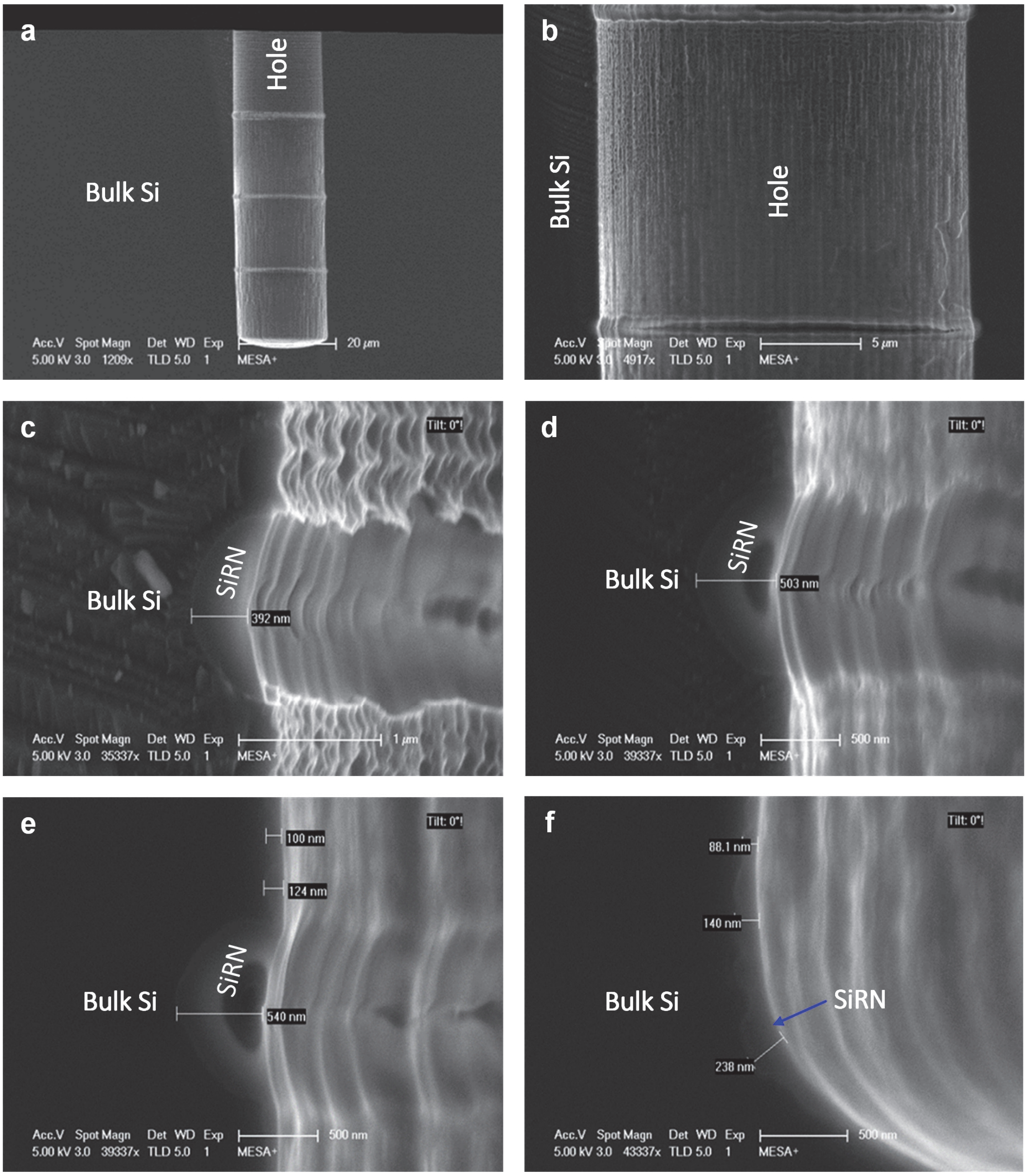

Fig. S4 Structural profile after etching in phosphoric acid (85 wt% at 180ºC) with an etching rate of around 4.47 nm min^{-1}. **a**) An overview. **b**) zoom in of the transition region, **c** the remaining SiRN layer at the first semicircular gap, **d**) the second semicircular gap, **e**) the third semi-circular gap, and **f**) at the bottom corner of the high aspect ratio microhole.

SI5. Temperature dependence of thermal oxidation profile

It is known that the thermal oxidation profile depends strongly on the temperature and shape of the underneath substrate. At low temperature (≤ 900ºC), the grown oxide is thinner at edges than at the flat surfaces, and thinnest at the corners[1,2]. Therefore, "sharp tips" can be formed at convex corners, as illustrated in Fig. S5a, which may fail if an etching step is applied subsequently. Oxide growth at a higher temperature (≥ 1050ºC) is rather conformal at convex silicon corners[2,3], as shown in Fig. S5b and S5c.

For LOCOS, a SiRN layer was deposited as a structural material before the thermal oxidation, which forms eventually an inversion mask after timed etching of SiRN. It is known that the etching rate of SiRN is significantly reduced when the film is annealed at 1100ºC[4]. Considering this, 1050ºC was used as oxidation temperature for LOCOS, as the oxide layer formed at this temperature is relatively conformal at convex silicon corners, and it does not reduce the etching rate significantly.

For digital etching of polysilicon, thermal oxidation was performed at 1050ºC for two times to avoid the presence of sharp concave corners at the outer surface of the oxide layer, as shown in the blue circle in Fig. S5b. This can be problematic for the nitride removal as corner lithography may also occur at these positions. By stripping the layer resulting from the first oxidation, a smoothed sidewall profile of high aspect ratio microholes can be obtained, which can inhibit the formation of sharp concave corners in the second oxidation, as shown in Fig. S5d.

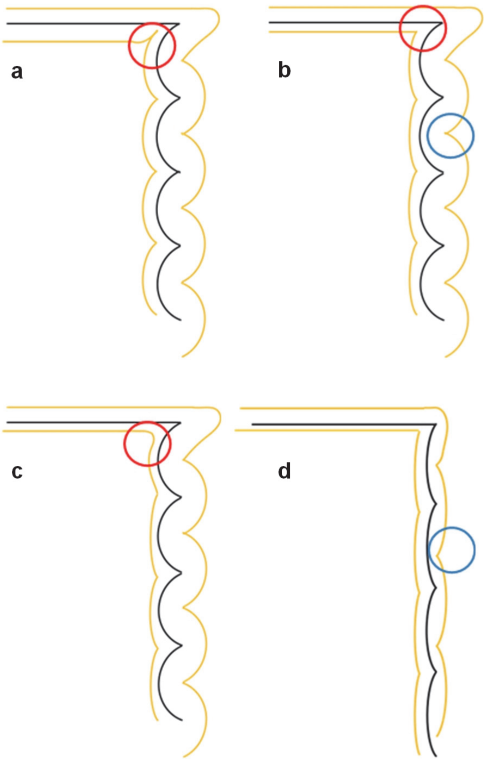

Fig. S5 Illustration of thermal oxidation profile at different temperatures. The yellow lines indicate the boundary of the oxide layer. **a**) Thermal oxidation profile at 900ºC, with a "sharp tip" formed at the convex silicon corner indicated by the red circle. **b**) Thermal oxidation profile at 1050ºC: the outer surface of the oxide layer shows sharp corners (blue circle) but the inner surface is quite smooth. **c**) Thermal oxidation profile at 1100ºC. **d**) Structure after second run of oxidation at 1050ºC: both the inner and outer surface of the oxide layer are relatively smooth.

References

1 Hirose, K., Shiraishi, F. & Mita, Y. A simultaneous vertical and horizontal self-patterning method for deep three-dimensional microstructures. *J. Micromech. Microeng.* **17**, S68–S76 (2007).

2 Kim, G. M. *et al.* Replication molds having nanometer-scale shape control fabricated by means of oxidation and etching. *J. Nanosci. Nanotech.* **2**, 55–59 (2002).

3 Marcus, R. B. & Sheng, T. T. The oxidation of shaped silicon surfaces. *J. Electrochem. Soc.* **129**, 1278–1282 (1982).

4 Milek, J. T. Silicon nitride for microelectronic applications: part 1 preparation and properties. *Springer Science & Business Media* (2013).
